# m^6^A RNA Methylation Regulators Impact Prognosis and Tumor Microenvironment in Renal Papillary Cell Carcinoma

**DOI:** 10.3389/fonc.2021.598017

**Published:** 2021-03-16

**Authors:** Lianze Chen, Baohui Hu, Xinyue Song, Lin Wang, Mingyi Ju, Zinan Li, Chenyi Zhou, Ming Zhang, Qian Wei, Qiutong Guan, Longyang Jiang, Ting Chen, Minjie Wei, Lin Zhao

**Affiliations:** ^1^ Department of Pharmacology, School of Pharmacy, China Medical University, Shenyang, China; ^2^ Liaoning Key Laboratory of Molecular Targeted Anti-Tumor Drug Development and Evaluation, Liaoning Cancer Immune Peptide Drug Engineering Technology Research Center, Key Laboratory of Precision Diagnosis and Treatment of Gastrointestinal Tumors, Ministry of Education, China Medical University, Shenyang, China; ^3^ Liaoning Medical Diagnosis and Treatment Center, Shenyang, China

**Keywords:** epigenetic modification, m^6^A RNA methylation, tumor microenvironment, prognostic signature, renal papillary cell carcinoma

## Abstract

Accumulating evidence has proven that N6-methyladenosine (m^6^A) RNA methylation plays an essential role in tumorigenesis. However, the significance of m^6^A RNA methylation modulators in the malignant progression of papillary renal cell carcinoma (PRCC) and their impact on prognosis has not been fully analyzed. The present research set out to explore the roles of 17 m^6^A RNA methylation regulators in tumor microenvironment (TME) of PRCC and identify the prognostic values of m^6^A RNA methylation regulators in patients afflicted by PRCC. We investigated the different expression patterns of the m^6^A RNA methylation regulators between PRCC tumor samples and normal tissues, and systematically explored the association of the expression patterns of these genes with TME cell-infiltrating characteristics. Additionally, we used LASSO regression to construct a risk signature based upon the m^6^A RNA methylation modulators. Two-gene prognostic risk model including IGF2BP3 and HNRNPC was constructed and could predict overall survival (OS) of PRCC patients from the Cancer Genome Atlas (TCGA) dataset. The prognostic signature-based risk score was identified as an independent prognostic indicator in Cox regression analysis. Moreover, we predicted the three most significant small molecule drugs that potentially inhibit PRCC. Taken together, our study revealed that m^6^A RNA methylation regulators might play a significant role in the initiation and progression of PRCC. The results might provide novel insight into exploration of m^6^A RNA modification in PRCC and provide essential guidance for therapeutic strategies.

## Introduction

Renal cell carcinoma (RCC) is one of the most prevalent malignant disease originating from renal tubular epithelium, represent 2–3% of all human cancers. Renal papillary cell carcinoma is the second most common pathological subtype of renal cell carcinoma, accounting for 18.5% (1–3). PRCC is classified by molecular subtypes into type 1 and type 2. Type 2 PRCC tumors are more aggressive and have a worse prognosis compared with clear cell renal cell carcinoma (ccRCC) (4). Treatment of advanced RCC relies on targeted drugs, for example, sunitinib, sorafenib, axitinib, pazopanib, cabozantinib, and lenvatinib, but has limited efficacy in the treatment of PRCC (5). PRCC and ccRCC are not identical in terms of pathological mechanisms, and PRCC does not account for a high proportion of RCC, leading to its exclusion in large clinical trials of certain drugs (6). PRCC has not received effective attention and the research progress is slow. Although PRCC patients can be diagnosed and operated on with ultrasound at an early stage, the current use of targeted therapy drugs is not effective, leaving many PRCC patients missing out on the best treatment opportunities. Therefore, seeking novel targets and prognostic biomarkers of PRCC is of profound significance.

N6-methyladenosine (m^6^A) is a methylation modification that can occur on RNA adenine (A) (7). It is one of the most common modification in mRNA, rRNA, tRNA, microRNA, and long non-coding RNA (8). m^6^A dynamics and functions are executed by three groups of proteins: Methyltransferases or “writers,” demethylases or “erasers,” and m^6^A -binding proteins or “readers” (9). Writers mainly include METTL3, METL14, KIA1429, WTAP, RBM15, and ZC3H13, they are in charge of RNA methylation (10). The demethylases, known as the “erasers,” can specifically target RNA m^6^A and mainly include ALKBH5 and FTO (11, 12). The readers are responsible for binding m^6^A sites and play specific regulatory roles for modified-RNA, including YTHDC1, YTHDC2, YTHDF1, YTHDF2, IGFBP1, IGFBP2, IGF2BP3, RBMX, and HNRNPC (13). m^6^A methylation regulates various aspect of RNA metabolism including abundance, alternative splicing, stability, nuclear export, decay, and translation (14). The deregulation of m^6^A regulators leads to decreased proliferation, self-renewal, survival, as well as differentiation (15). There is increasing evidence that the miscegenation of m^6^A RNA methylation regulators plays an important role in the occurrence and development of various tumors, such as liver cancer (16, 17), glioblastoma (18), osteosarcoma (19), and colorectal cancer (20). However, the relationship between m^6^A RNA methylation regulators and various clinicopathological features is still not fully clear.

In this study, we analyzed the expression of 17 m^6^A RNA methylation regulators in PRCC and investigated the relationship between m^6^A RNA methylation regulators and clinicopathologic characteristics of PRCC patients using transcriptome sequencing (RNA-seq) data downloaded from the TCGA database. Then we applied the CIBERSORT algorithm to further analyze the effect of m^6^A methylation regulator on the composition of PRCC immune-related cells. We found that m^6^A RNA methylation modulator played an important role in the progression of PRCC. Two m^6^A methylation regulators were screened to construct risk profiles to classify the prognosis of PRCC. Finally, we further analyzed the two powerful independent prognostic m^6^A methylation modulators and found that they played an important role in the malignant progression of PRCC.

## Methods

### Data Collection and Processing

The RNA-seq transcription data and corresponding clinical information of PRCC samples were downloaded from TCGA data portal (https://tcga-data.nci.nih.gov/tcga/), and the expression of these RNA-seq data was normalized by expectation-maximization (RSEM). A total of 289 PRCC cases and 32 adjacent normal tissues were included in the current study. This study meets the publication guidelines provided by TCGA Publication Guidelines, ethics committee approval was deemed not required.

### Detection of m^6^A RNA Methylation Regulators and Differential Expression Analysis

Based on previously published literature and PRCC gene expression data provided by TCGA, a total of 17 regulatory factors of m^6^A RNA methylation were identified. These 17 m^6^A regulators included six writers (METTL3, METL14, KIA1429, WTAP, RBM15, and ZC3H13) (10), two erasers (FTO and ALKBH5) (11, 12), and nine readers (YTHDC1, YTHDC2, YTHDF1, YTHDF2, IGFBP1, IGFBP2, IGF2BP3, RBMX, and HNRNPC) (13). PPI network analysis was performed at the STRING database (https://string-db.org) (21). PPI network analysis was performed at the STRING database (https://string-db.org) (22). *P*-value < 0.05 and | log2 fold change (FC)| ≥ 2 were set as the cutoff.

### Unsupervised Clustering for 17 m^6^A Regulators and Functional Annotation

To comprehensively illustrate the biological characteristics of the m^6^A regulators in PRCC, we performed the “ConsensusClusterPlus” package (50 iterations, sample rate of 80%) to classify the patients afflicted by PRCC into different subtypes (23). Subsequently, we conducted Gene Ontology (GO) and The Kyoto Encyclopedia of Genes and Genomes (KEGG) pathway enrichment analyses to functionally detect the difference on biological process between distinct m^6^A expression patterns using the “clusterProfiler” package (24). Gene set enrichment analysis (GSEA) was performed to investigate the hallmarks of tumor sets in different PRCC subtypes (http://software.broadinstitute.org/gsea/msigdb/index.jsp) (25).

### Evaluation of Tumor Immune Cells Infiltrating in Papillary Renal Cell Carcinoma

We used CIBERSORT algorithm, a bioinformatic algorithm, to estimate 22 types tumor-infiltrating immune cells in tumors, by characterizing the cell composition of complex tissues based on normalized gene expression profiles (26). Gene Set Enrichment Analysis (GSEA) was performed using GSEA 2.0.9 (http://www.broadinstitute.org/gsea/) (27).

### Development of the m^6^A Regulators-Related Prognostic Signature

Univariate Cox regression analysis was used to evaluate the prognostic value of m^6^A RNA methylation regulator. LASSO regression was used to further narrow the genes for prediction of the OS. Finally, we used the obtained prognostic genes to construct the risk score function to calculate for each patient. It calculated according to the following formula:

$$\text{Risk score}\;\text{= }\sum_{i=1}^{\text{n}}\beta \text{i}\times \text{Xi}$$

where βi is the coefficient and xi is the z-score-transformed relative expression value of each selected gene. All PRCC patients were assigned into two groups (a high-risk group and a low-risk group) according to the median risk score. We used the Kaplan-Meier method with the log-rank test to determine the difference in OS rates between two groups, and the package “survival ROC” within the R programming environment was used to plot Receiver Operating Characteristic (ROC) curves (28). The heat map was used to illustrate the difference in gene expression between high-risk and low-risk groups. TISIDB (http://cis.hku.hk/TISIDB) was used to study the influence of prognostic genes on the stage, molecular typing and immune typing of PRCC patients (29). Cox regression analysis was assessed to identify independent predictors of outcome in molecular pathological features.

### Identification of Potential Small Molecular Drugs

Potential drugs for the treatment of PRCC were selected using the Connectivity Map (CMap) database (https://portals.broadinstitute.org/CMap/) (30). We divided the differentially expressed genes from the high-risk and low-risk groups into up-regulated and down-regulated groups, and uploaded them into the CMap database for genomic enrichment analysis. In the obtained small molecule data results, the mean value closer to +1 indicated that the small molecule could promote the PRCC gene expression, while the mean value closer to −1 indicated that the small molecule might have an effect on inhibiting the progression PRCC. We screened the small drug molecules with enrichment value < 0 and *P* < 0.001, and accessed PubChem (http://www.pubchem.ncbi.nlm.gov) to analyze their 3D Conformer, a public repository of small molecules in properties (31).

### Statistical Analyses

Data analysis was performed with GraphPad Prism 7.0 (GraphPad, San Diego, CA, USA). Non-parametric Spearman rank correlation analysis was performed to calculate the correlation coefficient. One-way ANOVA was used to conduct different comparisons. Clinical pathological characteristics were compared between groups using Pearson’s Chi-square test. All the tests were two-sided, and significance was assumed for *P* < 0.05. All statistical analysis was performed by software R (version3.6.3).

## Results

### Expression Patterns of m^6^A Methylation Regulators in Papillary Renal Cell Carcinoma

**Figure 1:**
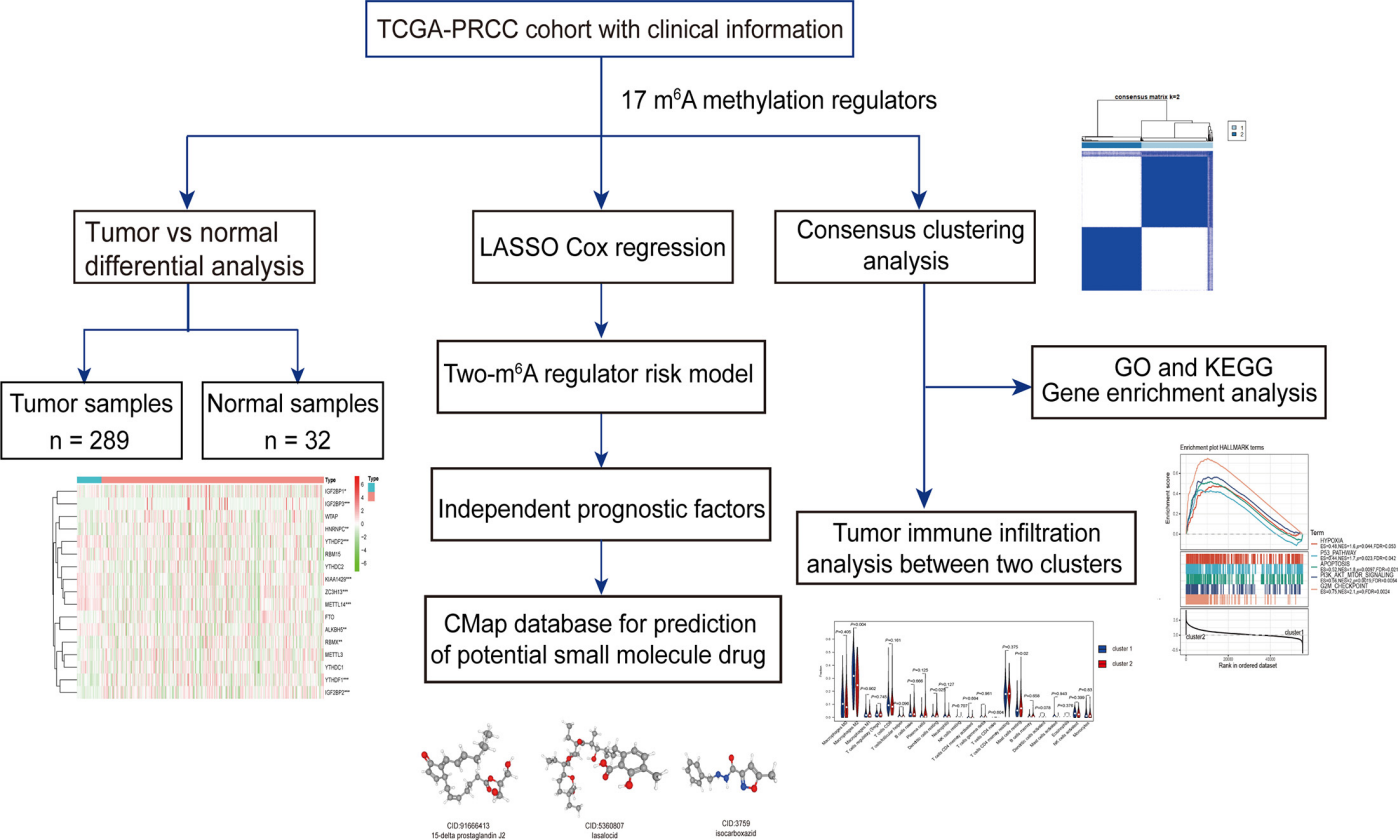
Workflow of this study.

**Figure 2:**
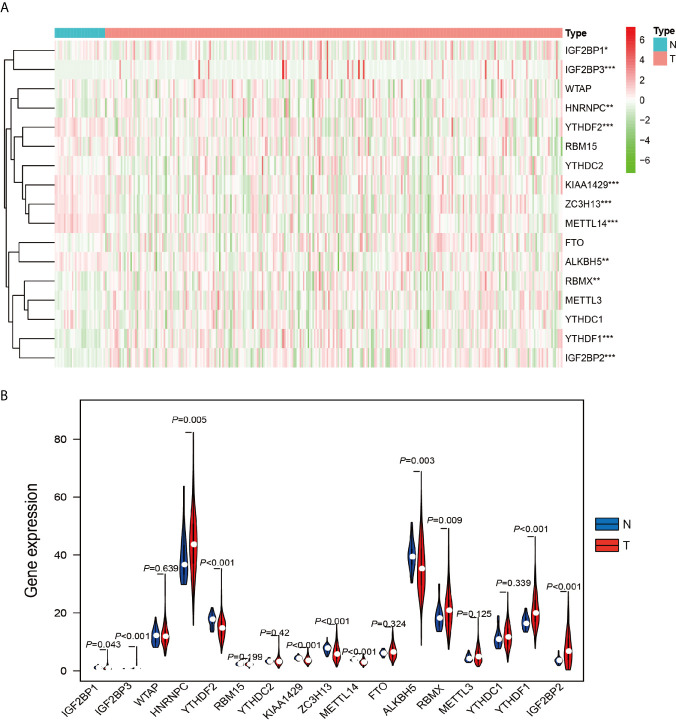
Differential expression of m^6^A RNA methylation regulators in tumor and adjacent normal tissues. **(A)** Heatmaps visualizing the expression levels of m^6^A RNA methylation regulators in each sample. **(B)** m^6^A RNA methylation regulators expressed differentially between tumor tissues and adjacent normal tissues. *P < 0.05, **P < 0.01, and ****P < 0.0001.

The work flowchart is displayed in **Figure 1**. The detailed clinicopathological information of these patients was listed in **Table 1**. Heatmap plots analysis was used to compare m^6^A RNA methylation regulators expression in tumor and normal tissues (**Figure 2A**). Compared to normal tissue samples, the expression levels of *RBMX*, *YTHDF1*, *IGF2BP3*, *IGF2BP2*, and *HNRNPC* were significantly increased in tumor tissue samples, while the expression of *KIAA1429*, *YTHDF2*, *ZC3H13*, *METTL14*, *IGF2BP1*, and *ALKBH5* in normal tissues was significantly higher than that in PRCC tissues (**Figure 2B**). Moreover, the interacting proteins of m^6^A RNA methylation regulators were analyzed using the STRING protein interaction online database. We found that the genes displayed significantly correlated expression patterns. In comparison with readers, the correlation among writers is notably stronger (**Figure S1A**). Additionally, using “corrplot” package in R software for further analysis, the results showed that the correlation between *METTL3* and *YTHDC1* was high, and *RBMX* was significantly in correlation with most of m^6^A RNA methylation regulators except the level of *IGF2BP1* and *ALKBH5* (**Figure S1B**). Taken together, the results indicated that the cross-talk among the writers, readers, as well as erasers of RNA methylation act as essential biological roles in tumorigenesis of PRCC.

**Table 1:** Clinical pathological parameters of patients with PRCC in this study.

Clinical characteristics	TCGA (N = 289)		
	Number	Percentage (%)	Dead number
Age			
≤65	177	61.25	23
>65	109	37.72	17
Unknown	3	1.04	0
Gender			
Female	76	26.30	11
Male	213	73.70	29
Stage			
I	173	59.86	10
II	21	7.27	1
III	51	17.65	15
IV	15	5.20	9
Unknown	29	10.03	5
Stage M			
M0	95	32.87	11
M1	9	3.11	7
Mx	171	59.17	21
Unknown	14	4.84	1
Stage N			
N0	49	16.96	7
N1	24	8.30	13
N2	4	1.38	3
Nx	211	73.01	16
Unknown	1	0.35	1

### Consensus Cluster Analyses Were Performed With m^6^A RNA Methylation Modulator to Distinguish Different Subgroups With Different Prognosis

**Figure 3:**
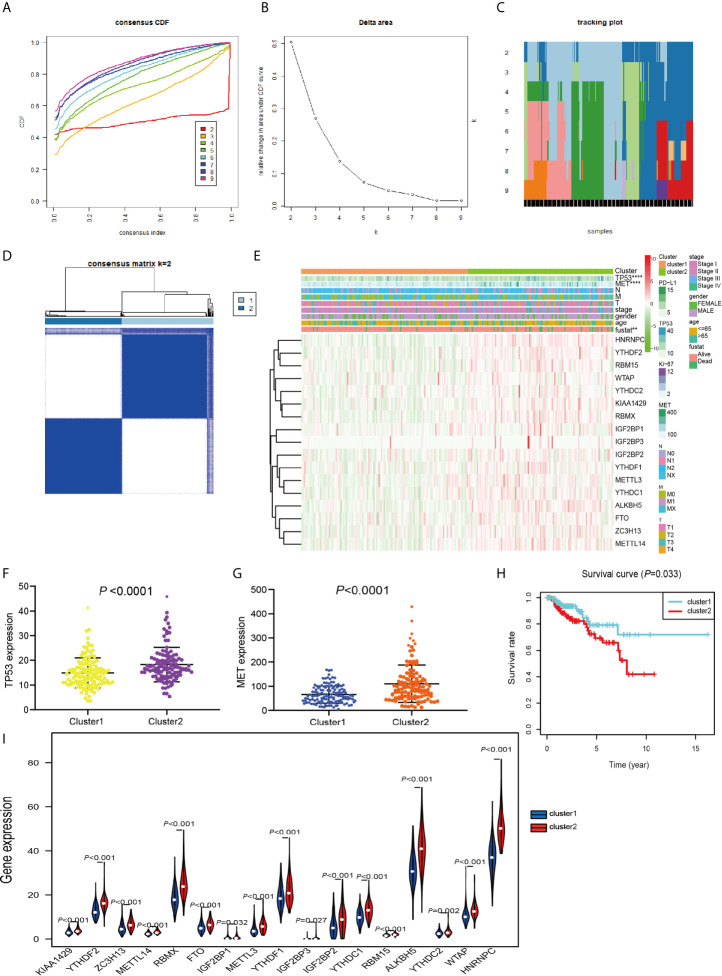
Differential clinicopathological features and OS of PRCC in the cluster 1/2 subgroups. **(A)** Consensus clustering cumulative distribution function (CDF) for k = 2 to 9. **(B)** Relative change in area under the CDF curve for k = 2 to 9. **(C)** The tracking plot for k = 2 to 9. **(D)** Consensus clustering matrix for PRCC. **(E)** Heatmap and clinicopathologic features of the two subgroups classified by the m^6^A RNA methylation regulatory gene consensus expression in PRCC. **(F)** Comparison of TP53 expressions in different subgroups. **(G)** Comparison of MET expressions in different subgroups. **(H)** Kaplan-Meier curves analysis for PRCC patients in cluster 1 and 2. **(I)** Violin plots displayed the distribution of m^6^A RNA Methylation Regulators expression in cluster 1 and cluster 2. **P < 0.01, ****P < 0.0001.

TCGA data consisting of 289 PRCC patients was performed consensus clustering based on the m^6^A RNA methylation regulator, using a class discovery tool “ConsensusClusterPlus.” Among k = 2 to 9, the results showed that the most stable clustering results can be obtained when k = 2. The result of consensus clustering indicated that 289 patients could be classified into two clusters with higher stability (**Figures 3A–D**). Next, we determined whether there was any significant difference in the clinicopathological characteristics and expression of several tumor-related markers between the two subgroups (**Figure 3E**). The results showed that no significant differences were noted in the clinical features between the two subgroups. More and more studies have revealed that the expression of *TP53* and *MET* plays an important role in PRCC research and we found that *TP53* and *MET* were highly expressed in cluster 2 (**Figures 3F, G**). Furthermore, cluster 2 patients had worse OS compared with patients within cluster 1 cohort (*P* = 0.033) (**Figure 3H**). Moreover, m^6^A RNA methylation regulators expression in cluster 2 was significantly higher than that in the cluster 1 (**Figure 3I**). These results indicated that the clustering subtypes identified by m^6^A regulators expression were closely associated with the heterogeneity of patients afflicted with PRCC.

### The Influence of m^6^A RNA Methylation Regulators Expression on the Biological Process and Signaling Pathways

**Figure 4:**
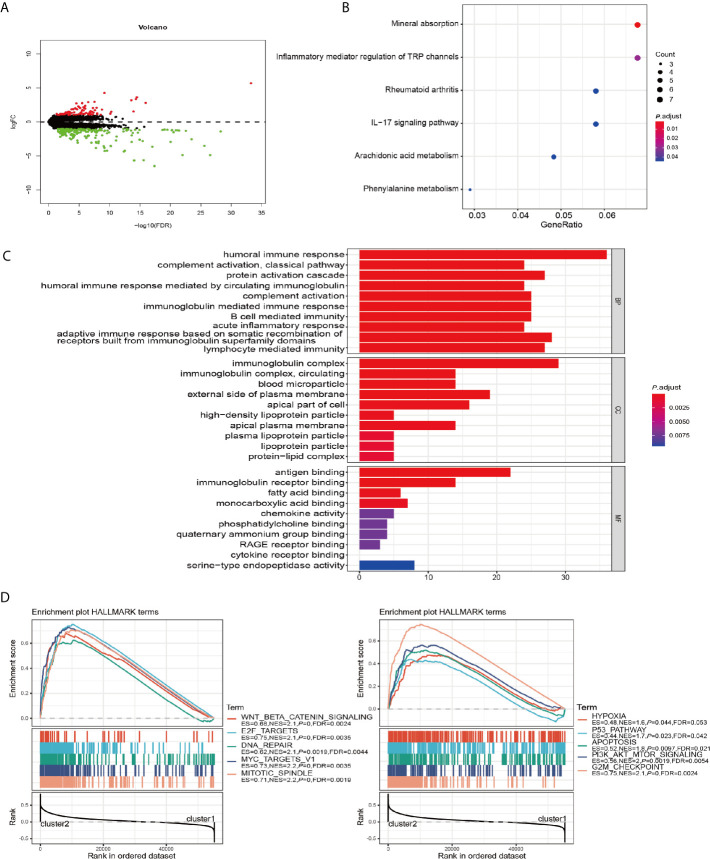
GO, KEGG, and GSEA analysis of DEGs. **(A)** Volcano plot showing differentially expressed genes between cluster 1 and cluster 2. **(B)** Functional annotation of the genes with different expression patterns between cluster 1 and cluster 2 using KEGG pathway terms. **(C)** Functional annotation of the genes with different expressions between cluster 1 and cluster 2 using GO terms. **(D)** GSEA showed that genes with higher expressions in cluster 2 were significantly enriched in hallmarks of malignant tumors.

To further explore the role of m^6^A RNA methylation regulators factors in the biological process and signaling pathways of PRCC, we carried out differential gene expression analysis between cluster 1 and cluster 2. Our results showed 176 significantly down and 88 up regulated mRNAs (**Figure 4A**). These differentially expressed genes (DEGs) were further analyzed by GO and KEGG pathway analyses. It was observed that biological pathways in KEGG were mainly enriched in inflammatory mediator regulation of TRP channels, rheumatoid arthritis, IL−17 signaling pathway, Arachidonic acid metabolism, and Phenylalanine metabolism (**Figure 4B**). GO analysis revealed the enrichment of terms related to humoral immune response, complement activation, acute inflammatory response, immunoglobulin complex, antigen binding, and fatty acid-binding among the top regulated categories. Most of the results showed that they correlated with immune response pathways (**Figure 4C**). Using GSEA to identify the potential different regulatory mechanisms between the two clusters, we found a number of hallmarks of malignant cancer including WNT β-catenin signaling, E2F targets, and DNA repair were significantly enriched in patients from cluster 2 cohort (**Figure 4D**). These data suggested a significant correlation between m^6^A methylation regulators and development of PRCC.

### Tumor Microenvironment Cell Infiltration Characteristics in Distinct m^6^A Modification Patterns

**Figure 5:**
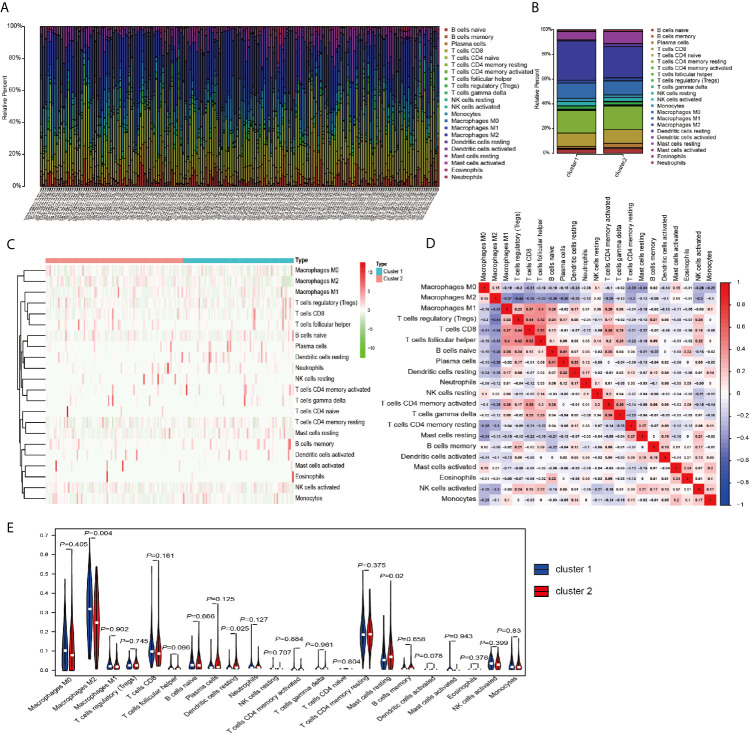
TME cell infiltration characteristics and transcriptome traits in distinct m^6^A modification patterns. **(A)** The summary of immune infiltration of 22 immune cells subpopulations in 177 samples. **(B)** Comparison of the abundance of immune infiltration of 22 immune cell subsets in different subgroups. **(C)** Heatmap of the 22 immune cell proportions in cluster 1 and cluster 2. **(D)** Correlation matrix of all 22 immune cell proportions. **(E)** The violin plot of the 22 immune cell proportions between cluster 1 and cluster 2.

The above results in this study showed that the expression of m^6^A RNA methylation regulators in PRCC has a close correlation with immune response, therefore, we focused our further analysis on the TME cell infiltration characteristics in distinct m^6^A modification patterns. We focused our further analysis on the infiltration of TME in two different cluster modes and finally evaluated 22 different immune cell types in 289 samples using the CIBERSORT algorithm (**Figure 5A**). Excluding samples of CIBERSORT *P*-value >0.05, 177 samples were enrolled. The fraction of 22 subpopulations abundance of immune cells in patients from two clusters was displayed in **Figure 5B**. Unsupervised cluster stratification of 22 immune cells was displayed in **Figure 5C**. The proportions of different subpopulations of tumor-infiltrating immune cells were weakly to moderately correlated (**Figure 5D**). In addition, we calculated the median absolute score CIBERSORT gave for 22 cell types in each cluster. The results showed that the fraction of macrophages M2 was significantly higher in cluster 1 than that in cluster 2. However, dendritic cells resting and mast cells resting were remarkably higher in cluster 2 than that in cluster 1 (**Figure 5E**).

**Figure 6:**
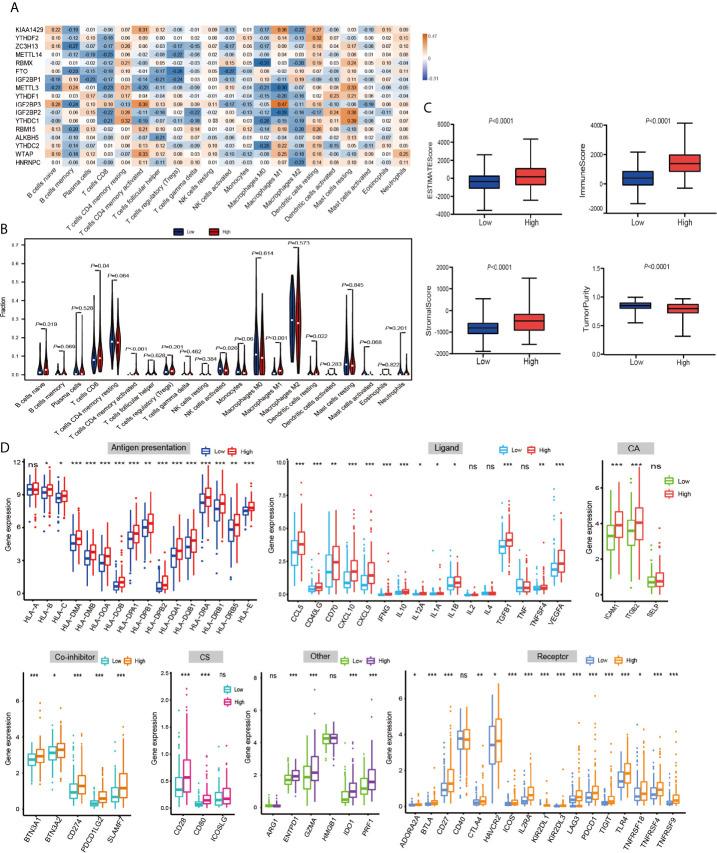
The relationship between the IGF2BP3 and the immune-related features in PRCC. **(A)** Spearman’s correlations between m^6^A RNA methylation regulators expression levels and immunomodulators. **(B)** The violin plot of the 22 immune cell proportions between high and low IGF2BP3 expression groups. **(C)** The difference in ESTIMATE scores, immune scores, and stroma scores between IGF2BP3 high expression and low expression subgroups. (*P* < 0.0001). **(D)** Effect of IGF2BP3 expression level on the expression of different immunomodulators. *P < 0.05, **P < 0.01, and ***P < 0.001.

Then, using a Spearman rank test, correlation analysis was performed between each type of immune cell and 17 m^6^A RNA methylation regulators (**Figure 6A**). IGF2BP3 caught our attention, and we found that it was significantly positively associated with many immune cells. Therefore, we further analyzed the overall infiltration of immune cells in patients with high and low expression of IGF2BP3 (**Figure 6B**). The results displayed that a fraction of B cells naive, T cells CD8, T cells CD4 memory activated, Macrophages M1, and Dendritic cells resting were significantly increased in the *IGF2BP3* high expression group. However, a fraction of NK cells activated was remarkably higher in low *IGF2BP3* expression groups than that in high *IGF2BP3* expression group. Next, we used ESTIMATE algorithm to score immune cell infiltration in patients with high and low expression of *IGF2BP3*. The results showed that high expression of *IGF2BP3* exhibited high ESTIMATE scores (*P* < 0.0001), immune scores (*P* < 0.0001), and stroma scores (*P* < 0.0001). However, the tumor purity scores in the high *IGF2BP3* expression patients were significantly lower than that in the low *IGF2BP3* expression patients (*P* < 0.0001), which meant that the TME with high expression pattern of *IGF2BP3* existed a dramatically increased immune cell infiltration, thus confirming the above findings (**Figure 6C**).

Immunomodulator agonists play an increasingly important role in the development of tumors. We therefore next investigated the impact of *IGF2BP3* on the expression of these immunomodulator agonists. All PRCC patients were subsequently divided into two groups (high expression group and low expression group) according to the *IGF2BP3* expression. Compared with the low *IGF2BP3* expression group, we found that these immunomodulator agonists’ expression was significantly higher in the high *IGF2BP3* expression group (**Figure 6D**). Specifically, programmed cell death 1 (PD-1), programmed cell death ligand 1 (PD-L1), programmed cell death 1 ligand 2 (PD-L2), and cytotoxic T-lymphocyte associated protein 4 (CTLA-4) also positively corelated with *IGF2BP3* expressions. In summary, we observed that IGF2BP3 is closely related to immunomodulatory factors in PRCC.

### Prognostic Value Screening of m^6^A RNA Methylation Regulators and Risk Signature Built

**Figure 7:**
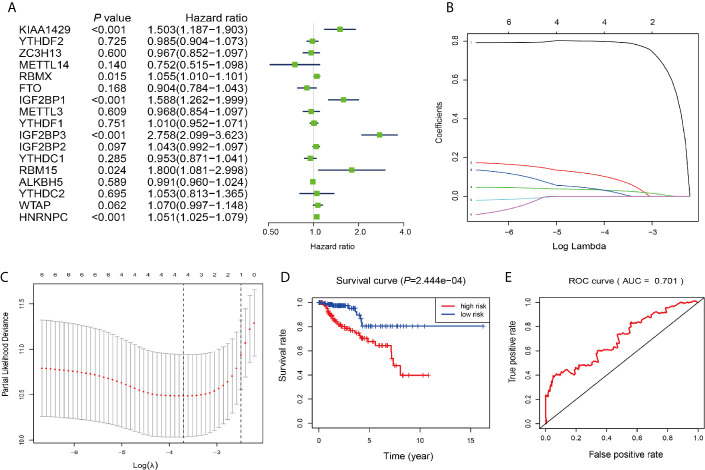
Construction of the risk signature according to the m^6^A RNA methylation regulators. **(A–C)** The process of building the signature containing two m^6^A RNA methylation regulators. Hazard ratios (HR, the center of the box) and 95% confidence intervals (CI, horizontal line) were calculated with Cox’s regression models. **(D)** Kaplan-Meier survival curves for OS in the CCRT-along groups of low and high risk. **(E)** ROC curve and AUC value.

Next, we investigated these m^6^A RNA methylation regulators for prognostic values on patient survival in PRCC patients. Univariate Cox regression analysis was performed to evaluate the relationship between the expression level of each m^6^A RNA methylation regulator and patients’ OS. The results demonstrated that KIAA1429 RBMX, IGF2BP1, IGF2BP3, RBM15, and HNRNPC were significantly correlated with OS with hard ratio (HR) larger than 1 (**Figure 7A**). In order to select the best prognosis-related genes, these six genes have undergone the LASSO regression analysis. Output of LASSO regression showed two regulators (IGF2BP3 and HNRNPC) were powerful prognostic factors.

Two genes (*IGF2BP3* and *HNRNPC*) were then applied to construct the risk signature. According to the coefficients of the LASSO selection in the Cox model, the risk scores were calculated and the high/low-risk groups were divided according to the median risk score in each dataset (**Figures 7B, C**). Survival was assessed in both the high-risk and low-risk groups, and the OS rate in the high-risk group was significantly lower than that in the low-risk group (**Figure 7D**). The area under the ROC curve (AUC) was 0.701 (**Figure 7E**), indicating the satisfactory accuracy of our two-m^6^A regulator signature for survival prediction of PRCC.

### Prognostic Risk Score and Two Prognostic Genes Showed Strong Associations With Clinicopathological Features in Papillary Renal Cell Carcinoma

**Figure 8:**
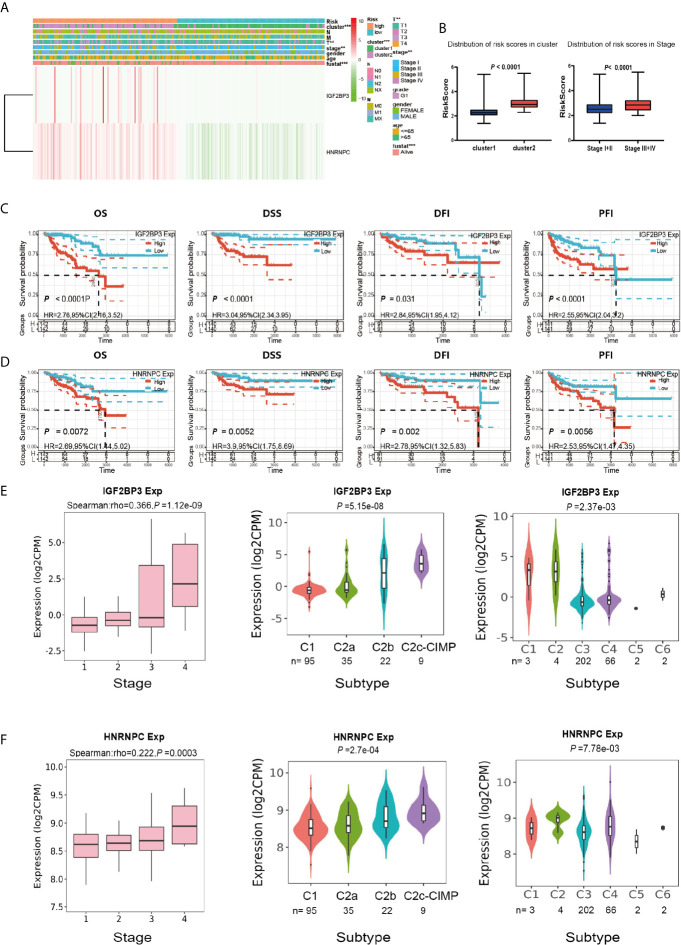
Prognostic risk scores and two prognostic genes showed strong associations with clinicopathological features in PRCC. **(A)** Heat map of the two gene expressions in the high-risk and low-risk groups. **P < 0.01, and ***P < 0.001. **(B)** The distribution of risk scores in different pathological stages and clusters. **(C)** The OS, DSS, RFS, and PFI based on IGF2BP3 expressions. **(D)** The OS, DSS, RFS, and PFI based on HNRNPC expressions. **(E)** The IGF2BP3 expression in different subtypes of the stage, molecular and immune. **(F)** The HNRNPC expression in different subtypes of the stage, molecular and immune.

We next sought to analyze the correlation between the risk score and the clinicopathological characteristics of PRCC patients. Heatmap plot showed that the gene expressions of *IGF2BP3* and *HNRNPC* were higher in the high-risk group than that in the low-risk group (**Figure 8A**). There were significant differences in cluster, pathological stage, and stage T between the high-risk group and the low-risk group (**Figure 8B**). To further investigate the clinical usefulness of *IGF2BP3* and *HNRNPC* expressions in PRCC, we compared the OS, disease-specific survival (DSS), recurrence-free survival (RFS), and platinum-free interval (PFI) based upon the expression of *IGF2BP3* and *HNRNPC*. The results showed that high expressions of *IGF2BP3* and *HNRNPC* were significantly correlated with shorter OS, DSS, RFS, and PFI of PRCC (**Figures 8C, D**). The *IGF2BP3* and *HNRNPC* expression in different subtypes of the stage, molecular, and immunity was analyzed using an integrated repository portal for tumor-immune system interactions (TISIDB). **Figures 8E, F** showed that the expression of *IGF2BP3* and *HNRNPC* increased along with stage I-IV, C2c-CIMP molecular subtypes, C1, and C2 immune subtypes. These results clearly showed the significant effect of *IGF2BP3* and *HNRNPC* expression on clinical outcome of patients with PRCC.

**Figure 9:**
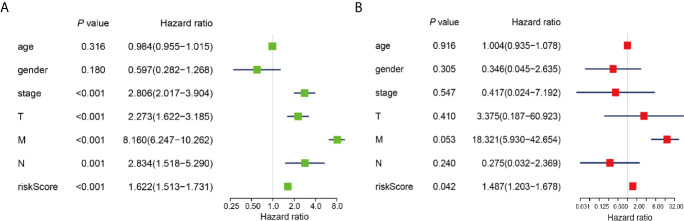
Identification of the independent prognostic factors in the PRCC cohort. **(A)** Single-factor analysis of clinicopathological parameter and risk score. **(B)** Multivariate analysis of clinicopathological parameters and risk score.

To determine whether risk signature and clinicopathological parameters were independent prognostic factors for PRCC, univariate and multivariate analysis was performed. Results of the univariate analysis showed that pathological stage, stage TNM, and risk score correlated with OS (**Figure 8A**). After adjusting for clinical and pathologic characteristics, we found that only the risk score associated with the OS rate of patients with PRCC (**Figure 8B**), indicating that our m^6^A regulator-related risk model was still a novel independent prognostic signature for predicting survival in patients with PRCC. Results of the univariate analysis showed that pathological stage, stage and risk score correlated with OS (**Figure 9A**). After adjusting for clinical and pathologic characteristics, we found that only the risk score associated with the OS rate of patients with PRCC (**Figure 9B**).

### Related Small Molecule Drugs Screening

**Figure 10:**
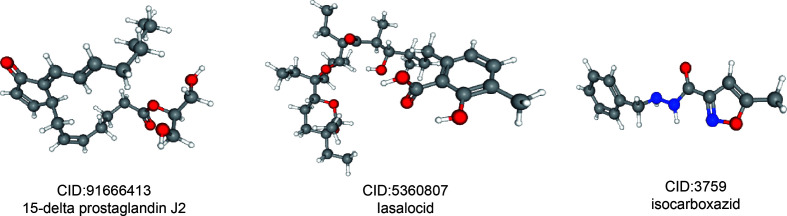
3D conformer of four most significant small molecule drugs.

In order to predict small molecule drugs that can inhibit PRCC, DEGs of high-risk and low-risk groups were assigned into up-regulated and down-regulated groups. Then we matched it to small molecule drug in the CMap database. Finally, we selected the three most important small molecular compounds (**Table 2**). The 3D structure of these three small molecular compounds were downloaded from the PubChem database (**Figure 10**). The negatively related molecular agents (enrichment < 0 and *P* < 0.05) for anti-PRCC were then identified. Based on the shear standard, total of three small-molecule compounds including 15-delta prostaglandin J2 (enrichment score = −0.587, *P* = 0), lasalocid (enrichment score = −0.864, *P* = 0.00062), isocarboxazid (enrichment score = −0. 781, *P* = 0.00088) were available that could be potential drugs for the treatment of patients afflicted with PRCC. These small molecules might potentially inhibit the occurrence of PRCC disease and provide recommendations for the selection of PRCC-targeted drugs, while the specific mechanism of action and effectiveness needs to be further studied.

**Table 2:** Four most significant small molecule drugs.

Rank	CMap name	Mean	N	Enrichment	*p*	CID
1	15-delta prostaglandin J2	−0.463	15	−0.587	0	91666413
2	lasalocid	−0.362	4	−0.864	0.00062	5360807
3	isocarboxazid	−0.387	5	−0.781	0.00088	3759

## Discussion

PRCC is a complex malignancy and the second most common RCC after CCRC (3). At present, studies on PRCC-related biomarkers are insufficient to meet the clinical requirements for patient diagnosis and prognosis. Moreover, there is a lack of knowledge about the use of PRCC-related mRNA as biomarkers and their internal interactions, so we need to conduct in-depth research on the occurrence and development of PRCC. Abnormal m^6^A RNA methylation has been shown to modulate the carcinogenic effects of many types of tumors. However, their roles in PRCC are unclear. In this study, we found that most m^6^A RNA methylation regulators were abnormally expressed in PRCC, and the PRCC cohort was divided into two subgroups according to the expression of m^6^A RNA methylation regulators, with significant differences in OS. The expression of 17 m^6^A methylation modulators may have important effects on the regulation of tumor markers such as MET and TP53 in PRCC patients. Besides, it is also closely related to the biological processes, key signaling pathways, and immune system of malignant PRCC. Therefore, to verify the correlation between 17 m^6^A methylation modulators and the immune system, CIBERSORT algorithm was used to further evaluate the infiltrated immune cells of the two subgroups. The proportion of immune cells varies significantly between samples. We then found that Cluster 1 had a significantly higher proportion of macrophage M2 than the Cluster 2 group. However, the proportion of dendritic cells and mast cells was significantly higher in Cluster 2. These results suggest that the expression of 17 m^6^A methylation modulators may affect the immune infiltration of PRCC patients, and it may have clinical significance for the characterization of individual difference. Next, prognostic risk characteristics were constructed based on IGF2BP3 and HNRNPC, and the patients were divided into high-risk and low-risk groups based on the median risk score.

As previously shown, abnormal expression of m^6^A RNA methylation modulators plays an important role in many types of cancer. The writer METTL3 and METTL14 were reported to promote tumorigenesis in hepatocellular carcinoma and acute myeloid leukemia (AML), but have the opposite effect in gastric cancer (18, 32). YTHDF1 can reflect the malignant degree of hepatocellular carcinoma and has prognostic value (33). High *ALKBH5* expression can promote proliferation of glial stem cell-like cells (34). WTAP can reposition METTL3 and METTL14 to their target RNA to enhance methyltransferase complex methylation activity (35). Interestingly, the reader YTHDF2 was found to inhibit invasion and migration in pancreatic cancer, but facilitate the migration of prostate cancer *in vitro* (36, 37). The m^6^A RNA modification, such as YTHDF1, was reported to regulate anti-tumor immune responses (16). Additionally, KIAA1429 is a critical methyltransferase that participates in the process of m^6^A modification. It has been reported that KIAA1429 mediated the m^6^A methylation of its direct downstream target GATA binding protein (GATA3), and thereby facilitating the malignant phenotypes of hepatoma cells (38). Moreover, KIAA1429 acts as an oncogenic role by regulating cyclin-dependent kinase (CDK1) in a m^6^A-independent manner in breast cancer (39). IGF2BP1 has been traditionally regarded as an oncogene and potential therapeutic target for cancers as well. Previous research has illustrated that the expression of *IGF2BP1* promotes hepatocellular carcinoma cell proliferation, migration, and invasion, and correlates with poor survival rate (40). The similar trend was also identified in non-small cell lung cancer, and high expression level of *IGF2BP1* facilitates the disease progression (41). However, there is still a lack of systematic researches on m^6^A methylation regulators for PRCC. By applying survival analysis, six m^6^A regulators including KIAA1429 and IGF2BP1 were found to exhibit significant correlation with the prognosis of PRCC as risk genes with HR > 1, indicating that these genes might contribute to the development of PRCC. Our research may also help offer a foundation for revealing the oncogene roles of the m^6^A regulators in PRCC for future study.

Furthermore, we found that the m^6^A methylation regulator high expression group (Cluster 2) patients had poorer OS, and most of the differential genes were enriched in immune-related signaling pathways, suggesting that the m^6^A methylation regulator expression is closely related to the prognosis and immunity of PRCC patients. One of the main findings of the current study is the establishment of risk characteristics of IGF2BP3 and HNRNPC, which could effectively predict the prognosis of PRCC cohort with the later stage having a higher risk score. Other than that, we found that the expression of *IGF2BP3* and *HNRNPC* was significantly increased in PRCC patients with later stage and C2c-CIMP subtypes. So far, no effective treatment for advancing PRCC has been found. And that type 2 papillary RCC is a heterogeneous disease with multiple distinct subgroups (42). The most distinct of the three Type 2 subgroups was that defined by the CpG island methylator phenotype (CIMP), which associated with the worst overall survival. Therefore, subtype classification of PRCC type 2 by specific molecular markers may provide important diagnosis. Further study of IGF2BP3 and HNRNPC may provide a new research direction for targeted therapy of PRCC.

We’ve learned from the previous research that IGF2BP3 promotes the proliferation of breast cancer cells by promoting the expression of TRIM25 (43). High expression of *IGF2BP3* can promote the invasiveness of colorectal cancer cells *in vitro* and *in vivo* (44). Overexpression of *IGF2BP3* mRNA and protein increases IGF2 translation and IGF1 receptor (IGF1R) signaling through PI3K and MAPK cascade reaction and promotes proliferation, invasion, and transformation of thyroid cancer cells (45). Increasing evidence has shown that the interaction between m^6^A modification and a variety of m^6^A modulators plays an important role in immune and inflammatory responses. In this study, we took the median expression level of *IGF2BP3* at the critical point and divided PRCC patients into two groups. The results showed that the high-expressed group showed stronger immune cell invasion compared with the low-expressed group, for example, B cells naive, T cells CD8, T cells CD4memory activated, Macrophages M1, and Dendritic cells significantly increased, which confirmed that the high-expressed *IGF2BP3* group had stronger antitumor immune activity. We examined the expression levels of some immunomodulator agonists in the *IGF2BP2* high expression group and the low expression group, and found that most of the *IGF2BP3* high expression group had higher expression levels of immunomodulator agonists than the low expression group, and the difference between the two groups was significant, especially the current PD‐1, PD-L1, PD‐L2, and CTLA‐4, which were closely related to PRCC research. These results indicate that high expression of *IGF2BP3* can better respond to anti-PD-L1 immunotherapy because the expression level of PD-L1 tends to be positively correlated with immunotherapy responsiveness (46).

It has been reported that over expression of *HNPNPC* genes may induce the recruitment of DNA repair factors in oxaliplatin-induced DNA double-strand breaks (47). *HNRNPC* expression levels in highly invasive GBM cell lines were increased and correlated with tumor grade (48). HNRNPC promotes breast cancer cells proliferation as well as tumor growth, and controls functions in endogenous dsRNA as well as downstream interferon response (49). Other studies have shown that HNRNPC can help advance OSCC through EMT (50). HNRNPC can help establish alternative cutting and polyadenylation profile of metastatic colon cancer cells (51). In our study, HNRNPC can predict the independent prognosis of PRCC and is associated with grading, while the specific ways and biological processes of RNA methylation regulation remain to be further studied. Both HNRNPC and IGF2BP3 found in this study are readers, and the specific mechanism of regulating PRCC still needs to be further verified by rigorous experiments.

In addition, we identified three potential small molecule drugs including 15-Delta prostaglandin J2, lasalocid, and Icarboxazid that might reverse poor prognosis of PRCC by using the CMap database. Among these drugs, 15-delta prostaglandin J2 has been found to suppress proliferation and arrest cell cycle in S-phase in human oral squamous cell carcinoma (CA9-22) cells (52). Additionally, 15-Delta prostaglandin J2 can suppress NF-κB and AP-1-mediated MMP-9 expression, and invasion of breast cancer cell by means of a heme oxygenase-1-dependent mechanism (53). It has been reported that Lasalocid mediates prostate cancer cells cell cycle arrest in G0/G1 phase by reducing G1 phase dependent proteins, and it can exert antitumor effect with production of reactive oxygen species (ROS) as well as mitochondrial hyperpolarization (54). Moreover, previous study has demonstrated that isocarboxazid inhibits the production of 6AcHA *in vivo*, thus supporting the involvement of MAO in HMBA metabolism (55). However, the effect and safety these small molecules drugs on PRCC are still lacking. Therefore, further study is urgently needed to reveal the potential of these drugs in PRCC treatment.

There are also some limitations to this study. First, we did not find other databases containing clinical survival data for verification. Second, this study is a computational study with no clinically relevant experiments to confirm it. Third, the data in our study were mainly Americans, so there may be a risk of selection bias conclusion.

## Conclusion

In summary, our study suggests that expression of 17 m^6^A RNA methylation modulators is closely related to the malignant progression and immunity of PRCC, and is highly correlated with the biological processes and pathways that promote malignant tumors. In addition, we identified strong prognostic markers significantly associated with poor clinical outcomes in PRCC and established risk characteristics that may help physicians more accurately estimate individual survival predictions and provide important guidance strategies for treatment selection.

## Data Availability Statement

Publicly available datasets were analyzed in this study. These data can be found here: https://portal.gdc.cancer.gov/.

## Author Contributions

LC and BH analyzed the data and drafted the manuscript. XS, LW, and MJ helped in interpreting the data. MZ, CZ, QW, LJ, and TC prepared all the figures. ZL and QG edited all the tables. LZ and MW designed the study. All authors contributed to the article and approved the submitted version.

## Funding

This work was supported by grants from the National Natural Science Foundation of China (Nos. 81573462, 81903658, and 81703560), Liaoning Revitalization Talents Program (No. XLYC1807201), Major Special S&T Projects in Liaoning Province (2019JH1/10300005), Liaoning Province Scientific Research Foundation (ZF2019038), and Shenyang S&T Projects (No. 19-109-4-09).

## Conflict of Interest

The authors declare that the research was conducted in the absence of any commercial or financial relationships that could be construed as a potential conflict of interest.

## References

[1] Bray F Ferlay J Soerjomataram I Siegel RL Torre LA Jemal A. Global cancer statistics 2018: GLOBOCAN estimates of incidence and mortality worldwide for 36 cancers in 185 countries. CA Cancer J Clin (2018) 68(6):394–424. 10.3322/caac.21492 30207593

[2] Jonasch E Gao J Rathmell WK. Renal cell carcinoma. BMJ (Clinical Res ed) (2014) 349:g4797. 10.1136/bmj.g4797 PMC4707715 25385470

[3] Moch H Cubilla AL Humphrey PA Reuter VE Ulbright TM. The 2016 WHO Classification of Tumours of the Urinary System and Male Genital Organs-Part A: Renal, Penile, and Testicular Tumours. Eur Urol (2016) 70(1):93–105. 10.1016/j.eururo.2016.02.029 26935559

[4] Steffens S Janssen M Roos FC Becker F Schumacher S Seidel C. Incidence and long-term prognosis of papillary compared to clear cell renal cell carcinoma–a multicentre study. Eur J Cancer (Oxford Engl 1990) (2012) 48(15):2347–52. 10.1016/j.ejca.2012.05.002 22698386

[5] Escudier B Porta C Schmidinger M Rioux-Leclercq N Bex A Khoo V. Renal cell carcinoma: ESMO Clinical Practice Guidelines for diagnosis, treatment and follow-up. Ann Oncol (2016) 27(suppl 5):v58–68. 10.1093/annonc/mdw328 27664262

[6] Courthod G Tucci M Di Maio M Scagliotti GV. Papillary renal cell carcinoma: A review of the current therapeutic landscape. Crit Rev Oncol/Hematol (2015) 96(1):100–12. 10.1016/j.critrevonc.2015.05.008 26052049

[7] Dubin DT Taylor RH. The methylation state of poly A-containing messenger RNA from cultured hamster cells. Nucleic Acids Res (1975) 2(10):1653–68. 10.1093/nar/2.10.1653 PMC343535 1187339

[8] Dominissini D Moshitch-Moshkovitz S Schwartz S Salmon-Divon M Ungar L Osenberg S. Topology of the human and mouse m6A RNA methylomes revealed by m6A-seq. Nature (2012) 485(7397):201–6. 10.1038/nature11112 22575960

[9] Yang Y Hsu PJ Chen YS Yang YG. Dynamic transcriptomic m(6)A decoration: writers, erasers, readers and functions in RNA metabolism. Cell Res (2018) 28(6):616–24. 10.1038/s41422-018-0040-8 PMC5993786 29789545

[10] Scholler E Weichmann F Treiber T Ringle S Treiber N Flatley A. Interactions, localization, and phosphorylation of the m(6)A generating METTL3-METTL14-WTAP complex. RNA (2018) 24(4):499–512. 10.1261/rna.064063.117 29348140 PMC5855951

[11] Tang C Klukovich R Peng H Wang Z Yu T Zhang Y. ALKBH5-dependent m6A demethylation controls splicing and stability of long 3’-UTR mRNAs in male germ cells. Proc Natl Acad Sci United States America (2018) 115(2):E325–e33. 10.1073/pnas.1717794115 PMC5777073 29279410

[12] Ding C Zou Q Ding J Ling M Wang W Li H. Increased N6-methyladenosine causes infertility is associated with FTO expression. J Cell Physiol (2018) 233(9):7055–66. 10.1002/jcp.26507 PMC13482491 29384212

[13] Wojtas MN Pandey RR Mendel M Homolka D Sachidanandam R Pillai RS. Regulation of m(6)A Transcripts by the 3’–>5’ RNA Helicase YTHDC2 Is Essential for a Successful Meiotic Program in the Mammalian Germline. Mol Cell (2017) 68(2):374–87.e12. 10.1016/j.molcel.2017.09.021 29033321

[14] Sun T Wu R Ming L. The role of m6A RNA methylation in cancer. Biomed Pharmacother = Biomed Pharmacother (2019) 112:108613. 10.1016/j.biopha.2019.108613 30784918

[15] Liu N Pan T. N6-methyladenosine-encoded epitranscriptomics. Nat Struct Mol Biol (2016) 23(2):98–102. 10.1038/nsmb.3162 26840897

[16] Zhao X Chen Y Mao Q Jiang X Jiang W Chen J. Overexpression of YTHDF1 is associated with poor prognosis in patients with hepatocellular carcinoma. Cancer Biomark (2018) 21(4):859–68. 10.3233/CBM-170791 PMC13078334 29439311

[17] Cheng X Li M Rao X Zhang W Li X Wang L. KIAA1429 regulates the migration and invasion of hepatocellular carcinoma by altering m6A modification of ID2 mRNA. OncoTargets Ther (2019) 12:3421–8. 10.2147/OTT.S180954 PMC6510231 31118692

[18] Cui Q Shi H Ye P Li L Qu Q Sun G. m(6)A RNA Methylation Regulates the Self-Renewal and Tumorigenesis of Glioblastoma Stem Cells. Cell Rep (2017) 18(11):2622–34. 10.1016/j.celrep.2017.02.059 PMC5479356 28297667

[19] Miao W Chen J Jia L Ma J Song D. The m6A methyltransferase METTL3 promotes osteosarcoma progression by regulating the m6A level of LEF1. Biochem Biophys Res Commun (2019) 516(3):719–25. 10.1016/j.bbrc.2019.06.128 31253399

[20] Li T Hu PS Zuo Z Lin JF Li X Wu QN. METTL3 facilitates tumor progression via an m(6)A-IGF2BP2-dependent mechanism in colorectal carcinoma. Mol Cancer (2019) 18(1):112. 10.1186/s12943-019-1038-7 31230592 PMC6589893

[21] Szklarczyk D Franceschini A Wyder S Forslund K Heller D Huerta-Cepas J. STRING v10: protein-protein interaction networks, integrated over the tree of life. Nucleic Acids Res (2015) 43(Database issue):D447–52. 10.1093/nar/gku1003 PMC4383874 25352553

[22] Robinson MD McCarthy DJ Smyth GK. edgeR: a Bioconductor package for differential expression analysis of digital gene expression data. Bioinf (Oxford England) (2010) 26(1):139–40. 10.1093/bioinformatics/btp616 PMC2796818 19910308

[23] Wilkerson MD Hayes DN. ConsensusClusterPlus: a class discovery tool with confidence assessments and item tracking. Bioinformatics (2010) 26(12):1572–3. 10.1093/bioinformatics/btq170 PMC2881355 20427518

[24] Yu G Wang LG Han Y He QY. clusterProfiler: an R package for comparing biological themes among gene clusters. OMICS (2012) 16(5):284–7. 10.1089/omi.2011.0118 PMC3339379 22455463

[25] Subramanian A Tamayo P Mootha VK Mukherjee S Ebert BL Gillette MA. Gene set enrichment analysis: a knowledge-based approach for interpreting genome-wide expression profiles. Proc Natl Acad Sci U S A (2005) 102(43):15545–50. 10.1073/pnas.0506580102 PMC1239896 16199517

[26] Newman AM Liu CL Green MR Gentles AJ Feng W Xu Y. Robust enumeration of cell subsets from tissue expression profiles. Nat Methods (2015) 12(5):453–7. 10.1038/nmeth.3337 PMC4739640 25822800

[27] Yoshihara K Shahmoradgoli M Martinez E Vegesna R Kim H Torres-Garcia W. Inferring tumour purity and stromal and immune cell admixture from expression data. Nat Commun (2013) 4:2612. 10.1038/ncomms3612 24113773 PMC3826632

[28] Hanley JA McNeil BJ. The meaning and use of the area under a receiver operating characteristic (ROC) curve. Radiology (1982) 143(1):29–36. 10.1148/radiology.143.1.7063747 7063747

[29] Ru B Wong CN Tong Y Zhong JY Zhong SSW Wu WC. TISIDB: an integrated repository portal for tumor-immune system interactions. Bioinf (Oxford England) (2019) 35(20):4200–2. 10.1093/bioinformatics/btz210 30903160

[30] Lamb J Crawford ED Peck D Modell JW Blat IC Wrobel MJ. The Connectivity Map: using gene-expression signatures to connect small molecules, genes, and disease. Science (2006) 313(5795):1929–35. 10.1126/science.1132939 17008526

[31] Li Q Cheng T Wang Y Bryant SH. PubChem as a public resource for drug discovery. Drug Discov Today (2010) 15(23-24):1052–7. 10.1016/j.drudis.2010.10.003 PMC3010383 20970519

[32] Wang S Sun C Li J Zhang E Ma Z Xu W. Roles of RNA methylation by means of N(6)-methyladenosine (m(6)A) in human cancers. Cancer Lett (2017) 408:112–20. 10.1016/j.canlet.2017.08.030 28867248

[33] Zhou Y Yin Z Hou B Yu M Chen R Jin H. Expression profiles and prognostic significance of RNA N6-methyladenosine-related genes in patients with hepatocellular carcinoma: evidence from independent datasets. Cancer Manage Res (2019) 11:3921–31. 10.2147/CMAR.S191565 PMC6503205 31118805

[34] Chai RC Wu F Wang QX Zhang S Zhang KN Liu YQ. m(6)A RNA methylation regulators contribute to malignant progression and have clinical prognostic impact in gliomas. Aging (2019) 11(4):1204–25. 10.18632/aging.101829 PMC6402513 30810537

[35] Ping XL Sun BF Wang L Xiao W Yang X Wang WJ. Mammalian WTAP is a regulatory subunit of the RNA N6-methyladenosine methyltransferase. Cell Res (2014) 24(2):177–89. 10.1038/cr.2014.3 PMC3915904 24407421

[36] Chen J Sun Y Xu X Wang D He J Zhou H. YTH domain family 2 orchestrates epithelial-mesenchymal transition/proliferation dichotomy in pancreatic cancer cells. Cell Cycle (Georgetown Tex) (2017) 16(23):2259–71. 10.1080/15384101.2017.1380125 PMC5788481 29135329

[37] Li J Meng S Xu M Wang S He L Xu X. Downregulation of N(6)-methyladenosine binding YTHDF2 protein mediated by miR-493-3p suppresses prostate cancer by elevating N(6)-methyladenosine levels. Oncotarget (2018) 9(3):3752–64. 10.18632/oncotarget.23365 PMC5790497 29423080

[38] Lan T Li H Zhang DL Xu L Liu HL Hao XY. KIAA1429 contributes to liver cancer progression through N6-methyladenosine-dependent post-transcriptional modification of GATA3. Mol Cancer (2019) 18(1):186. 10.1186/s12943-019-1106-z 31856849 PMC6921542

[39] Qian JY Gao J Sun X Cao MD Shi L Xia TS. KIAA1429 acts as an oncogenic factor in breast cancer by regulating CDK1 in an N6-methyladenosine-independent manner. Oncogene (2019) 38(33):6123–41. 10.1038/s41388-019-0861-z 31285549

[40] Zhou X Zhang CZ Lu SX Chen GG Li LZ Liu LL. miR-625 suppresses tumour migration and invasion by targeting IGF2BP1 in hepatocellular carcinoma. Oncogene (2015) 34(8):965–77. 10.1038/onc.2014.35 24632613

[41] Kato T Hayama S Yamabuki T Ishikawa N Miyamoto M Ito T. Increased expression of insulin-like growth factor-II messenger RNA-binding protein 1 is associated with tumor progression in patients with lung cancer. Clin Cancer Res (2007) 13(2):434–42. 10.1158/1078-0432.CCR-06-1297 17255263

[42] Cancer Genome Atlas Research N Linehan WM Spellman PT Ricketts CJ Creighton CJ Fei SS. Comprehensive Molecular Characterization of Papillary Renal-Cell Carcinoma. N Engl J Med (2016) 374(2):135–45. 10.1056/NEJMoa1505917 PMC4775252 26536169

[43] Wang Z Tong D Han C Zhao Z Wang X Jiang T. Blockade of miR-3614 maturation by IGF2BP3 increases TRIM25 expression and promotes breast cancer cell proliferation. EBioMedicine (2019) 41:357–69. 10.1016/j.ebiom.2018.12.061 PMC6444029 30797711

[44] Xu W Sheng Y Guo Y Huang Z Huang Y Wen D. Increased IGF2BP3 expression promotes the aggressive phenotypes of colorectal cancer cells in vitro and vivo. J Cell Physiol (2019) 234(10):18466–79. 10.1002/jcp.28483 30895618

[45] Panebianco F Kelly LM Liu P Zhong S Dacic S Wang X. THADA fusion is a mechanism of IGF2BP3 activation and IGF1R signaling in thyroid cancer. Proc Natl Acad Sci U S A (2017) 114(9):2307–12. 10.1073/pnas.1614265114 PMC5338560 28193878

[46] Patel SP Kurzrock R. PD-L1 Expression as a Predictive Biomarker in Cancer Immunotherapy. Mol Cancer Ther (2015) 14(4):847–56. 10.1158/1535-7163.MCT-14-0983 25695955

[47] Hu W Lei L Xie X Huang L Cui Q Dang T. Heterogeneous nuclear ribonucleoprotein L facilitates recruitment of 53BP1 and BRCA1 at the DNA break sites induced by oxaliplatin in colorectal cancer. Cell Death Dis (2019) 10(8):550. 10.1038/s41419-019-1784-x 31320608 PMC6639419

[48] Park YM Hwang SJ Masuda K Choi KM Jeong MR Nam DH. Heterogeneous nuclear ribonucleoprotein C1/C2 controls the metastatic potential of glioblastoma by regulating PDCD4. Mol Cell Biol (2012) 32(20):4237–44. 10.1128/MCB.00443-12 PMC3457347 22907752

[49] Wu Y Zhao W Liu Y Tan X Li X Zou Q. Function of HNRNPC in breast cancer cells by controlling the dsRNA-induced interferon response. EMBO J (2018) 37(23):e99017. 10.15252/embj.201899017 30158112 PMC6276880

[50] Huang GZ Wu QQ Zheng ZN Shao TR Chen YC Zeng WS. M6A-related bioinformatics analysis reveals that HNRNPC facilitates progression of OSCC via EMT. Aging (2020) 12(12):11667–84. 10.18632/aging.103333 PMC7343469 32526707

[51] Liu X Liu L Dong Z Li J Yu Y Chen X. Expression patterns and prognostic value of m(6)A-related genes in colorectal cancer. Am J Trans Res (2019) 11(7):3972–91. PMC6684930 31396313

[52] Hall JA Rusten M Abughazaleh RD Wuertz B. Effects of PPAR-γ agonists on oral cancer cell lines: Potential horizons for chemopreventives and adjunctive therapies. Head Neck (2020) 42(9):2542–54. 10.1002/hed.26286 PMC7657659 32519370

[53] Jang HY Hong OY Youn HJ Kim MG Kim CH Jung SH. 15d-PGJ2 inhibits NF-κB and AP-1-mediated MMP-9 expression and invasion of breast cancer cell by means of a heme oxygenase-1-dependent mechanism. BMB Rep (2020) 53(4):212–7. 10.5483/BMBRep.2020.53.4.164 PMC7196191 31964465

[54] Kim KY Kim SH Yu SN Park SG Kim YW Nam HW. Lasalocid induces cytotoxic apoptosis and cytoprotective autophagy through reactive oxygen species in human prostate cancer PC-3 cells. Biomed Pharmacother = Biomed Pharmacother (2017) 88:1016–24. 10.1016/j.biopha.2017.01.140 28178613

[55] Conley BA Sewack GF Egorin MJ Subramanyam B Page JG Grieshaber CK. The effect of the monoamine oxidase inhibitor isocarboxazid on the canine metabolism of the cell-differentiating agent hexamethylene bisacetamide. Cancer Chemother Pharmacol (1991) 28(1):33–8. 10.1007/BF00684953 2040031

