# Supplementary figures and images for: m6A RNA Methylation Regulators Impact Prognosis and Tumor Microenvironment in Renal Papillary Cell Carcinoma
Source: Front Oncol. 2021 Mar 16;11:598017. doi: 10.3389/fonc.2021.598017 (PMC8008109; doi:10.3389/fonc.2021.598017)

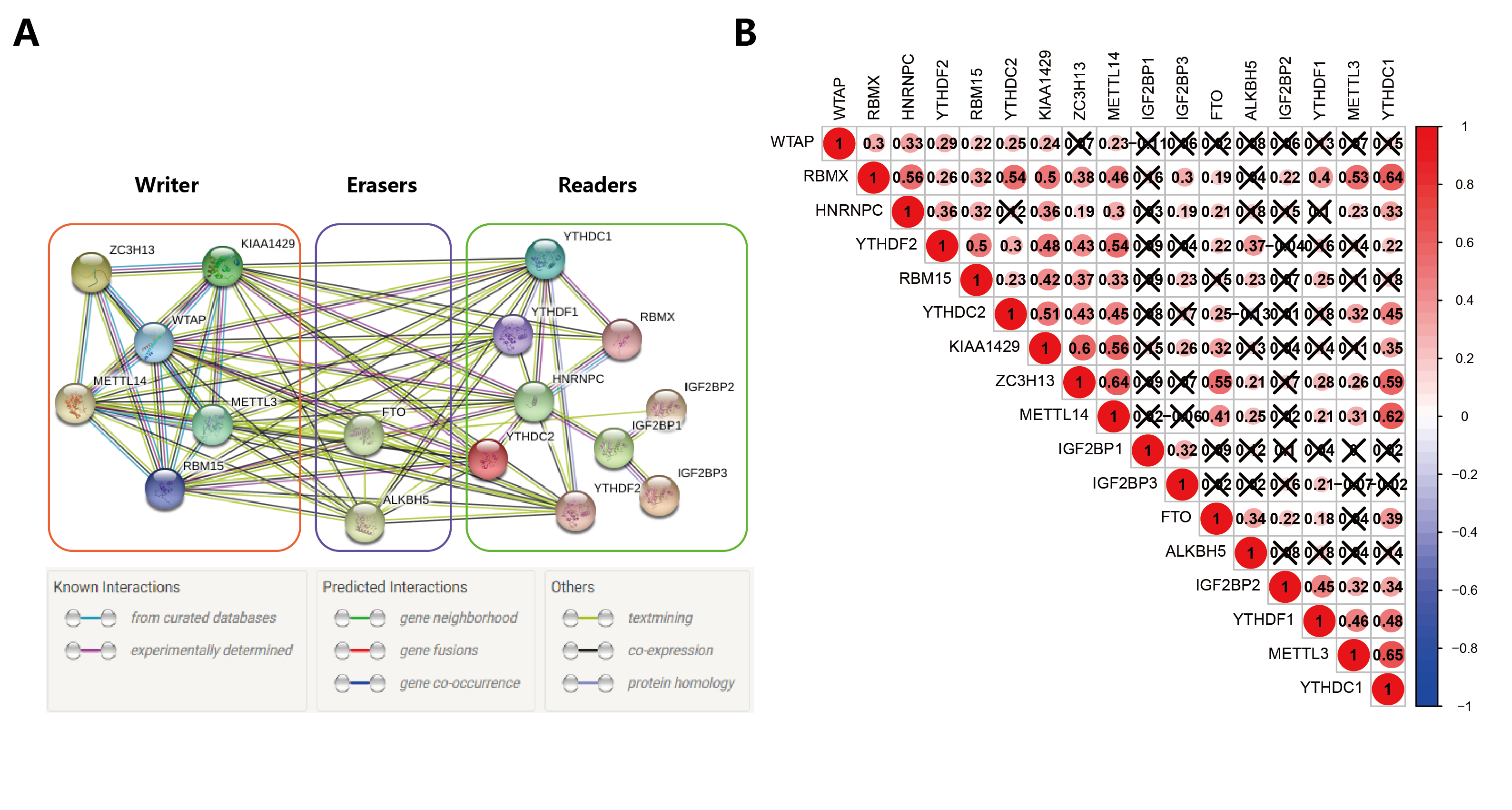

Supplement: Supplementary file 1 [file Image_1.tif]
